# Health Research Ethics in Southern Africa: Building Capacity and Cultivating Excellence

**DOI:** 10.1177/15562646251347549

**Published:** 2025-06-30

**Authors:** Zaynab Essack, Paul Ndebele, Heidi Matisonn, Jantina de Vries, Keymanthri Moodley, Stuart Rennie, Joseph Ali, Nancy Kass, Carel IJsselmuiden, Nhlanhla Mkhize, Mariana Kruger, Ann Strode, Warren Freedman, Douglas Wassenaar

**Affiliations:** 1 South African Research Ethics Training Initiative (SARETI), School of Applied Human Sciences, University of KwaZulu-Natal, Pietermaritzburg, South Africa; 2 Centre for Community-Based Research, 56867 Human Sciences Research Council, Sweetwaters, South Africa; 3 Department of Global Health, Milken Institute School of Public Health, George Washington University, Washington DC, USA; 4 The EthicsLab, Department of Medicine, 37716 University of Cape Town, Rondebosch, Western Cape, South Africa; 6 Division of Medical Ethics and Law, Department of Medicine, Faculty of Medicine and Health Sciences, 26697 Stellenbosch University, Cape Town, South Africa; 7 Department of Social Medicine, 2331 University of North Carolina at Chapel Hill, Chapel Hill, North Carolina, USA; 8 UNC Center for Bioethics, Department of Social Medicine, 2331 University of North Carolina at Chapel Hill, Chapel Hill, North Carolina, USA; 9 Bloomberg School of Public Health, 1466 Johns Hopkins University, Baltimore, Maryland, USA; 10 Berman Institute of Bioethics, 1466 Johns Hopkins University, Baltimore, Maryland, USA; 11 Council on Health Research for Development, Geneva, Switzerland; 12 Department of Pediatrics and Child Health, Faculty of Medicine and Health Sciences, 26697 Stellenbosch University, Stellenbosch, South Africa

**Keywords:** Bioethics, research ethics, & RCR, research ethics committee/IRB review, ethics education, developing countries

## Abstract

The health research landscape in southern Africa is becoming increasingly complex as research efforts intensify to address the region's significant disease burden. The increasing volume and complexity of health research in low- and middle-income countries (LMICs) highlights the ongoing need for enhanced research ethics capacity. To supplement a review published in 2014, this paper provides an overview of research ethics capacity-building initiatives supported through substantive long-term competitive awards from the Fogarty International Center of the US National Institutes of Health between 2014 and 2024. These programs aimed to enhance the capabilities of research ethics committees (RECs) and strengthen research ethics capacity throughout Southern Africa, with broader benefits to the African continent and globally as collaborative health research increases. The programs have successfully developed and delivered comprehensive research ethics curricula tailored to research ethics concerns in the region. Trainees and graduates include clinicians, researchers, REC members, REC administrators, lawyers, and ethicists, with focused efforts to ensure gender parity. This increased representivity of scholars has improved the membership on RECs and in skilled research ethics leadership in the region. For the benefit of future planning, this paper also describes some of the many challenges faced in delivering on program goals – including COVID-19. We also describe innovative solutions developed to address these challenges and meet the needs of students, faculty and institutions, while cultivating excellence in health research ethics. The paper concludes by highlighting areas for future research, underscoring the importance of continued diverse global investment in research ethics capacity to protect research participants and maintain and improve ethical standards and practice in health research within the region and globally. This will enable the development of innovative evidence-based global health solutions based on ethical research.

## Introduction

The health research landscape in southern Africa is becoming increasingly complex as research efforts intensify to address the region's significant disease burden (e.g., Ndembi et al., 2024). This diversity includes differences in the structure and functioning of research ethics committees (RECs) and ethics review processes, ranging from well-resourced, well-regulated RECs in some countries (Cleaton-Jones & Wassenaar, 2010) to poorly-resourced and poorly-regulated RECs, requiring much infrastructural and conceptual support in other countries (Milford et al., 2006; Silaigwana & Wassenaar, 2015). In the early 2000s, the Fogarty International Center (FIC) of the US National Institutes of Health (NIH) recognized this and initiated the competitive research ethics funding scheme upon which the programs described in this paper are based (Chaudhry et al., 2022; Horn, 2017; Millum et al., 2013; Mokgatla et al., 2018; Tangwa & Munung, 2022). RECs are pivotal in protecting research participants’ rights, welfare, and safety by ensuring adherence to ethical principles (Silaigwana & Wassenaar, 2015). However, RECs in southern Africa face various challenges. These include gaps in ethics capacity, funding, and operational constraints (Motari et al., 2015), as well as a lack of representivity in membership, among others. The over-representation of physicians and scientists and the under-representation of ethicists, lawyers, community representatives, and laypersons affect the comprehensiveness of ethics review and the protection of vulnerable populations (Chaudhry et al., 2022).

Rapid advancements in health research and emerging global health crises raise important ethical questions that go beyond the rights and responsibilities of research participants. For example, practices like non-invasive brain manipulation, coupled with evolving insights into the structure and functioning of the brain, raise important and novel questions about mental privacy and brain data confidentiality; while global research on artificial intelligence (AI) has highlighted how existing biases and discrimination are being programed into AI systems (Shaw et al., 2024). These scientific developments have changed the bioethics agenda and require a critical examination of the theories, methods, and approaches used in scholarship and educational practices.

To address these challenges, various organizations (e.g., Wellcome, FIC, and the US NIH Clinical Center for Bioethics) have made significant investments to strengthen the capacity for academic, intellectual, and research ethics review in the Global South (Horn, 2017; Mokgatla et al., 2018). This paper specifically focuses on the capacity-building programs in the southern African region funded by the NIH/FIC between 2014 and 2024 (including South Africa, Zimbabwe, Zambia, Botswana, Uganda and Madagascar). However, it is not a comprehensive description of all programs in the southern African region. This paper describes developments following those detailed in an earlier paper (Ndebele et al., 2014a) that describes related outputs for the period 2000–2012.

## Regional Health and Health Research Profile

The southern African region faces several shared health concerns. The region has a high prevalence of HIV and TB, with South Africa being one of the most affected countries globally (Atkinson & Mabey, 2019; Bates et al., 2015). Despite efforts to prevent HIV transmission and expand antiretroviral therapy, co-infection with TB remains a significant challenge. Malaria is also a significant health concern, posing substantial challenges to public health due to its high incidence and impact on communities (WHO, 2022b). Countries in this region, like others globally, are also facing an increasing burden of non-communicable diseases like hypertension, diabetes, and cardiovascular illness due to urbanization and lifestyle changes (Keates et al., 2017). Comprehensive global public health strategies are needed to prevent and manage these disease burdens (WHO, 2022a).

Ongoing pandemics such as cholera and COVID-19 also strain healthcare systems. COVID-19 specifically, raised important considerations for health research ethics, including ensuring equitable access to treatments and vaccines, protecting participants’ rights and well-being, and fostering trust in public health initiatives. During the pandemic, RECs encountered difficulties balancing urgent COVID-19 research with the need for rapid and rigorous ethics review. Moreover, they had to navigate rapid scientific advancements while tackling concerns such as informed consent and the fair allocation of research benefits (Burgess et al., 2023; Orievulu et al., 2024; Rossouw et al., 2021). These challenges highlight the need for appropriate research ethics frameworks and procedures to guide research during global public health emergencies (Burgess et al., 2023; Salamanca-Buentello et al., 2024).

## Regional Health Research Ethics System Capacity and Needs

Robust ethics and legal frameworks are fundamental to participant protection. Ethics guidelines are informed by international standards, such as the current versions of the Declaration of Helsinki, CIOMS and WHO guidelines, as well as by local research ethics guidelines. For example, South Africa's statutory National Health Research Ethics Council (NHREC) registers and regulates all South African RECs and provides comprehensive, legally binding research ethics guidelines (National Health Research Ethics Council, 2024 ). Similarly, in 2013, Zambia enacted a National Health Research Act that, for the first time, codified regulatory standards for health research in the country. Zimbabwe's National Guidelines for Conducting Health Research (Medical Research Council of Zimbabwe, 2011) and Good Clinical Practice Guidelines (Medicines Control Authority of Zimbabwe, 2022) detail protocols for ethical conduct, review processes, and the management of clinical trials, with a focus on participant protection and adherence to international standards.

Capacity building is essential for researchers, ethicists, and REC members to gain an understanding of these frameworks. This understanding enhances RECs’ ability to critically review, implement, and evaluate their recommendations (Essack & Wassenaar, 2018). Ensuring that effective oversight is in place throughout the research process is vital for upholding global ethical standards (Cleaton-Jones & Wassenaar, 2010; Silaigwana & Wassenaar, 2015; Strode et al., 2021).

Programs and initiatives supported by the NIH/Fogarty strengthen RECs in Africa through capacity-building in response to the increasing volumes and complexities of health research protocols and the need to integrate ethical considerations throughout the research process (Horn, 2017; Ndebele et al., 2014a). In addition to strengthening frameworks, increasing capacity in health research ethics more broadly is necessary to protect participants and communities as research complexities evolve. This includes the integration of new technologies like big data and AI, the need for rapid ethical decision-making in pandemics (Parker et al., 2020; Rossouw et al., 2021; Salamanca-Buentello et al., 2024; Smith & Miller, 2021; Tangwa & Munung, 2022) and the identification of community perspectives and values that can inform research practice (Koloi-Keaikitse et al., 2021; Slack et al., 2018; Zulu et al., 2022).

Research ethics infrastructure in the Southern African region is evolving. In 2017, South Africa had 30 RECs, Botswana had 4, and Zambia and Zimbabwe had 3 each (Mokgatla et al., 2018). While these numbers provide a historical reference, ongoing developments in health research ethics may have led to later improvements in REC structures and capacity in these countries.

## Program Descriptions and Rationale

### Fogarty International Centre (FIC) Bioethics Training Programs in Southern Africa

Since the early 2000s, FIC has provided substantial competitive support to successful international bioethics training programs, including several in southern Africa. This section discusses the programs since 2014 (cf. Ndebele et al., 2014a for a review of programs prior to 2014), emphasizing their impact in the region, and more broadly. Most of the programs are based in southern Africa, but given the collaborative nature of the awards, their reach is continental and global.

### South Africa

#### Master of Science in Global Health Ethics, University of Cape Town (UCT): 2022–2027

The Master of Science in Global Health Ethics offered by the EthicsLab in the Faculty of Health Sciences at UCT addresses the ethical challenges posed by rapid developments in neuroscience, AI, data science, and genomics. This program focuses on empowering students to understand, investigate, and challenge the political and economic arrangements that perpetuate power imbalances in the sciences and bioethics.

The program includes courses on the historical, social, and political factors shaping global health research, African intellectual histories and perspectives, methods and practices of global health research ethics, and the ethics of new and emerging health technologies. In the second year, students take courses on research with underrepresented communities and special topics in health research ethics (for example pandemics, planetary/one health). The program culminates in a minor dissertation, allowing students to both design rigorous ethics research and assess the quality of evidence in ethics research.

#### Master of Social Science (Health Research Ethics), South African Research Ethics Training Initiative (SARETI), University of KwaZulu-Natal (UKZN): 2000–2026

The South African Research Ethics Training Initiative (SARETI) program at the University of KwaZulu-Natal aims to build advanced capacity for the ethics review and implementation of health research in Africa. This section will describe SARETI I-III, and the current iteration of the program, SARETI IV.

#### SARETI I-III

SARETI I was the first Masters program in research ethics in Africa (Ndebele et al., 2014a), comprising a unique multi-institutional and pragmatic education and research program. SARETI I was initially an ethics major in the MPH degree at the University of Pretoria, before it became a MSocSc degree at UKZN. However, SARETI II (2007–2010, 5R25TW001599-09) and III (2012–2017, 5R25TW001599-17) were offered only as an MSocSc at UKZN largely due to a change of PI from the University of Pretoria (IJsselmuiden) to UKZN (Wassenaar), and in part due to lessons learned as the program evolved. SARETI II and III created many opportunities for graduates to build professional networks across Africa and globally. This resulted in an increasing number of staff and student publications, presentations, and research invitations, supported by formal collaboration with the Johns Hopkins Berman Institute of Bioethics and the HIV/AIDS Vaccines Ethics Group (http://www.saavi.org.za/haveg/). SARETI student interactions also led to the reduction of bottlenecks in African research systems in areas like analysis, quality improvement of REC administration and digitization. For example, some students focused on research ethics committee administrators (RECAs) to improve quality and efficiency of ethics review (Kasule et al., 2016). Digitization, harmonization and real-time quality control were the goals of RHInnO Ethics, an African-based REC platform developed by COHRED but now owned and managed by an African non-profit led by a SARETI III graduate, EthiXPERT (www.ethixpert.org.za), as was a pan-African assessment of research ethics systems through the SARETI-related MARC studies (Mokgatla, 2017, 2018). The absence of an African-authored research ethics textbook led to the publication of a freely available book (Kruger et al., 2014), spearheaded by SARETI faculty and graduates.

Between 2007 and 2020, SARETI II and III graduated 45 masters students from 13 African countries. Several SARETI masters students went on to complete PhDs elsewhere. From our post-training tracking, most of these graduates occupy positions of influence in research ethics development in Africa and in the United States. SARETI II and III did not support funding for PhD students because at the time, doctoral programs were not supported by FIC (see Ulrich et al., 2025 – this issue). Despite this, SARETI I did support one PhD to completion. However, increasing demand for doctoral-level training resulted in the current SARETI PhD Leadership Program (see Ulrich et al., 2025 – this issue) while the renewed Masters program with a legal emphasis continues at UKZN (see below) due to deliberate succession planning with colleagues. Importantly, we also invited selected SARETI I, II and III graduates to our SARETI African Health Research Ethics Symposium III symposium, at which significant time was allocated to identifying priorities which informed the design of our successful SARETI PhD application to FIC (see Ulrich et al., 2025– this issue).

Less visible, but of key importance, we believe, is that the core executive team of SARETI I, II, III and our current PhD has remained unchanged for 24 years to date, which provides us with the trust and international perspective to continue improving SARETI training and programming.

#### SARETI IV

SARETI IV, funded in 2021 (MPIs Strode and Essack), built on the academic framework provided by SARETI I-III and continues to address the increasing need for African expertise in research ethics, adding a special focus on enhancing legal capacity on RECs. The program equips early-to-mid-career individuals from across Africa with the academic and practical skills required to teach, supervise, and ethically review health research. The multidisciplinary Masters degree includes law modules, practical work with local RECs, and expanded offerings in social sciences and empirical bioethics. Graduates are anticipated to contribute to the ethical conduct and regulation of local and international health research in Africa, and enhance both the teaching and research landscape.

SARETI IV combines face-to-face modular coursework with practical work with local RECs and leading NIH-funded organizations such as the Centre for the AIDS Programme of Research in South Africa (CAPRISA) and the Human Sciences Research Council (HSRC). It also includes a research project on a health research ethics topic relevant to the student's home institution or country. Skills development workshops, structured mentoring support, individual coaching, and a virtual mentorship community support students throughout the program (cf. e.g., Munro et al., 2024). Students complete a capstone assessment to evaluate their assimilation of practical and theoretical knowledge through a mock REC activity. Top performers receive scholarships for research ethics workshops and/or international conferences and are appointed as honorary REC members for a year.

#### Program Deliverables

SARETI IV aims to graduate 20 Masters students, improve legal capacity on RECs, enhance capacity to review and undertake social science and biomedical research, and build a network of competent African and global colleagues able to reason critically. These outcomes are designed to ensure that graduates are well-equipped to contribute significantly to the ethical conduct and regulation of health research to international standards in their respective countries and institutions.

#### Advancing Research Ethics Training in Southern Africa (ARESA) Bioethics Leadership Program, Stellenbosch University and the Centre for Bioethics, University of North Carolina: 2011–2016

The ARESA (Advancing Research Ethics in Southern Africa) program comprised a comprehensive postgraduate diploma program in research ethics at Stellenbosch University in collaboration with the University of North Carolina at Chapel Hill. It succeeded the IRENSA program described in Ndebele et al. (2014a), and its primary goals were to equip students with in-depth knowledge of research ethics, provide customized mentorship, and offer practical experiences through REC observations and exercises. The program also developed research ethics materials for trainees’ home institutions. ARESA endeavored to foster a culture of ethically responsible research conduct in Southern Africa through network development, dissemination of information, public awareness, and outreach activities such as hosting an annual research ethics conference and publishing the ARESA newsletter.

ARESA specifically targeted mid-career professionals and REC members, focusing on those from diverse professional backgrounds across southern Africa. ARESA emphasized both empirical and conceptual research approaches, encouraging students to undertake practical assignments and participate in empirical projects. The ARESA program covered three modules on ethics, law, and human rights; the ethical imperative of dual review to ensure research meets standards in both the host and sponsor countries; and research and vulnerability. Each module involved intensive two-week training sessions. Students also completed a practicum assignment and were required to present their research ethics topics during in-person sessions.

#### Program Deliverables

Key deliverables included the graduation of students with a postgraduate diploma qualification, the development of research ethics course materials, personalized mentorship in grant writing and research methods, REC observations, and the creation of research ethics materials for home institutions. Network development through annual conferences and the establishment of the Research Ethics Committee Association of Southern Africa (REASA) which was intended to be an organization through which the ARESA graduates could continue their capacity development activities. Public awareness and outreach were supported through the ARESA newsletter and annual seminars, contributing to the development of a robust research ethics community in Southern Africa.

### Zimbabwe

#### United States-Zimbabwe Research Ethics Training Program (US-Zim RETP)

The United States-Zimbabwe Research Ethics Training Program (US-Zim RETP) was established in 2024 to address previous incidents of unethical research in Zimbabwe, probably due to a relative lack of research ethics training and inadequate oversight in Zimbabwe (Ndebele et al., 2014b). Its goal is to develop capacity for research ethics education, training, and ethics research in the country. The program is a collaborative effort between George Washington University, Milken Institute School of Public Health (GWSPH) in the United States and the University of Zimbabwe Faculty of Medicine and Health Sciences (UZFMHS).

The US-Zim RETP aims to transform research ethics education, training, and research not only at UZFMHS but also at other universities and research institutions in Zimbabwe. By doing so, it can improve the research ethics environment to support the growing emphasis on local and international health research in the country (Matimba et al., 2020; Ngwenya, 2021). This will be accomplished through the establishment of a blended teaching and learning program, including face-to-face and online instruction, for a Master's level degree in health research ethics. The program will also include attachments at various RECs throughout Zimbabwe, as well as workshops, seminars, short courses, and ethics research, and faculty include graduates of older FIC-funded training programs described elsewhere (Ndebele et al., 2014a)

The US-Zim RETP program will address several needs in research ethics in Zimbabwe. These include the necessity for research ethics oversight and research on ethics in response to the increasing quality and quantity of health research across institutions in the country (Mashaah et al., 2014; Shoko et al., 2018). Additionally, the program will address the need for international collaborative research aligned with research ethics standards, particularly in areas such as clinical trials, genomics, and biobanking. It will also focus on global health research, requiring robust risk-benefit analysis and ethics oversight that is contextualized to Zimbabwe.

Furthermore, the program will aim to align research ethics with local beliefs, norms, values, and socio-cultural sensitivities. It will enhance research on ethical issues in Zimbabwe and address the ethical considerations brought about by the COVID-19 pandemic (cf. Salamanca-Buentello et al., 2024), with the aim of preparing for future global pandemics. Finally, the program will contribute to building a pool of research ethics graduates in Zimbabwe, as well as training future educators for research ethics education in the country.

#### Program Deliverables

As set out above, the Masters program includes attachments at various RECs throughout Zimbabwe, workshops, seminars, short courses, and ethics research. The capacity-building program aims to strengthen research ethics education and capacity through various strategies. These include intensive faculty development activities to design ethics training curricula, establishing a two-year master's degree in Health Research Ethics at UZFMHS, and offering professional development courses through short-term training workshops and webinars. UZFMHS faculty members will participate in programs to enhance their research ethics teaching skills, working with experienced faculty from GWSPH and Africa-based research ethics experts.

Cultural sensitivity is a key aspect of the program's ethics training. It recognizes that certain research topics may conflict with local cultural norms, values, and beliefs. For example, discussing men who have sex with men is considered sensitive in Zimbabwe. Zimbabwean faculty will ensure that the course addresses cultural issues and other sensitivities within the local context. The program will equip trainees with the knowledge necessary to successfully conduct ethical research on such topics. To address the issue of cultural sensitivity, one of the required modules titled “Research ethics contextualized to the Zimbabwean culture and perspectives” specifically focuses on the application of global ethical principles in the Zimbabwean cultural context. It is expected that the module will equip fellows with skills to navigate ethical issues in the Zimbabwean context.

The program was recently awarded and is expected to produce outputs in the near future.

### Democratic Republic of Congo and Madagascar

#### Strengthening Bioethics Capacity and Justice in Health: 2004–2019

This training project sought to raise awareness, skills and local capacity in research ethics and bioethics among individuals and institutions involved in health promotion and education in Francophone Africa, focusing on the Democratic Republic of Congo and Madagascar. The core collaborative partnership comprised the University of North Carolina-Chapel Hill (USA), University of Namur (Belgium), the Kinshasa School of Public Health (DRC), and the Centre d’Infectiologie Charles Mérieux (Madagascar). Our approach centered on strengthening individual and institutional capacity, strongly linking research ethics with clinical and public health ethics, and embedding ethical issues in general with the socio-economic conditions commonly faced in low-income Francophone African settings.

**Program deliverables** included the development of a MPH program with a bioethics concentration at the Kinshasa School of Public Health (KSPH); the creation of a KSPH bioethics unit; customized training of selected Congolese and Malagasy health professionals and scholars; strengthening human resource capacity of local RECs; development of French-language bioethics curriculum materials; short-term research stays for scholars in Belgium and the United States; input into national research ethics guidance and regulations; grant-writing capacity building; bioethics and research ethics research and publications; and the conduct of workshops, seminars and short courses.

### Botswana, Uganda and Zambia

#### Johns Hopkins-Fogarty African Bioethics Training Program (2000–2025)

The Johns Hopkins-Fogarty African Bioethics Training Program (FABTP) began in 2000 (Ndebele et al., 2014a), providing certificate and master's level training in bioethics to promising scholars and practitioners from sub-Saharan Africa. In response to a changing bioethics environment in Africa, the program shifted focus in 2010 to invest in African *institutions* to deepen research ethics systems. It initiated three, year-long partnerships between JHU and the University of Zambia, the University of Botswana, and Makerere University (Uganda). Building on these partnerships and addressing common needs and complementary strengths, the African Bioethics Consortium was established. Through the consortium, JHU worked with the leadership of the three African universities – each including some FABTP alumni – to continue training strategically identified individuals from each institution in bioethics, and to work collaboratively on bioethics program building, developing strategic plans and working toward a sustainable bioethics presence at each university. Institutional leaders from the four consortium members met monthly virtually and annually in person; long- and short-term trainees underwent training in bioethics and research ethics at JHU in conjunction with trainees from each of the other two consortium institutions to build collaborative relationships; and consortium members visited each other's institutions, allowing learning exchanges in bioethics program building and IRB/REC enhancement to facilitate international best practice.

**Program deliverables** were identified at the level of the African Bioethics Consortium, and specific objectives were also established across consortium member institutions. Consortium-level goals included raising the profile of bioethics in sub-Saharan Africa; helping to establish bioethics units at African member institutions; sharing experiences and resources in bioethics; increasing the amount and quality of research in bioethics at member institutions; providing and supporting training in bioethics at institutions; and supporting the amount and quality of bioethics service at institutions.

All long-term trainees completed graduate coursework in research ethics, bioethics, and research methods at JHU*.* They also participated in IRB/REC observations, a weekly FABTP seminar, a weekly practicum tutorial, ethics seminars at JHU and the NIH, and regular individual meetings with JHU mentors. Trainees also completed funded practicum projects that were both consistent with their individual interests and of relevance and importance to their institution's strategic goals in bioethics/research ethics. After completing their JHU-based training, trainees returned to their home countries to conduct their practicum projects. Mentoring was provided by both JHU and partner African faculty. Short-term trainees, many of whom were consortium institution leaders, completed a one-month intensive global bioethics training program.

Ongoing mentoring of program alumni was also prioritized, with alumni engaged through regular communications, webinars and in-person reunion meetings. The program provided continuous mentoring by, for example, helping to support alumni-led scholarly research, writing and presentations (Table 1).

**Table 1.** Overview of FIC funded research ethics training programs in southern Africa.

Country	Grant Number	Year of funding	Awardee Institutions	Qualification type	Duration of program	Format of program
South Africa Stellenbosch PART TIME	Advancing Research Ethics in Southern Africa (ARESA); 1R25TW009722-01	2011–2016	Stellenbosch University, Division of Medical Ethics and Law; University of North Carolina-Chapel Hill, Center for Bioethics	Postgraduate Diploma	1 year	Intensive training modules, Practicum assignment, Annual ARESA seminar, ARESA newsletter
South Africa UKZN FULL TIME	South African Research Ethics Training Initiative II-III (SARETI); 1R25TW001599-01	2002–2019	University of KwaZulu-Natal, School of Applied Human Sciences	Master of Social Sciences (Health Research Ethics	2 years	Coursework, practicum assignments, workshops, site visits, thesis
South Africa UKZN FULL TIME	South African Research Ethics Training Initiative IV (SARETI); 1R25TW001599-01	2021–2026	University of KwaZulu-Natal, School of Applied Human Sciences	Master of Social Sciences (Health Research Ethics	2 years	Coursework, practicum assignments, REC observation, capstone project, workshops, research site visits, thesis
South Africa UCT FULL TIME	Master of Science in Global Health Ethics 5R25TW012219-03	2022–2027	University of Cape Town	Master of Science (Global Health Ethics)	2 years	Coursework, workshops, seminars, thesis
Zimbabwe FULL TIME	United States-Zimbabwe Research Ethics Training Program (US-Zim RETP); 1R25TW012713-01	2024–2029	The George Washington University, Milken Institute School of Public Health; University of Zimbabwe, Faculty of Medicine and Health Sciences	Master of Bioethics Education (MBE)	2 years	Workshops, seminars, attachments at RECs, annual conference/symposium
DRC and Madagascar FULL TIME	Building Bioethics Capacity and Justice in Health; R25TW007098	2004–2019	University of North Carolina-Chapel Hill; Kinshasa School of Public Health	MPH with concentration in bioethics	2 years	Workshops, short courses, seminars, research stays, individual mentoring, grant-writing support
Botswana, Zambia, Uganda FULL TIME	Johns Hopkins-Fogarty African Bioethics Training Program R25TW001604	2000–2025	Johns Hopkins University	Non-degree, Master's level	n/a	Strategic planning, short- and long-term training, consortium exchanges, workshops, resource exchange, research, targeted IRB strengthening

## Program Output Successes, Challenges, and Solutions to Date

### South Africa

SARETI and ARESA are the main focus in this next section, since UCT's accreditation is still ongoing and the formal program is yet to commence.

SARETI I-III has seen significant successes, including the graduation of trained professionals, the establishment of new research ethics curricula, and the development of a PhD program (see Ulrich et al., 2025, this issue) and networks and associations to promote ethical research practices of international standard. However, challenges such as funding constraints, logistical issues, and the need for ongoing mentorship and support remain. Solutions include leveraging online and blended learning approaches, securing additional funding sources, and strengthening collaborations with international partners.

#### SARETI IV:

##### Successes

The current active SARETI IV Masters program has made significant strides in achieving its goals. Notably, four students from the 2022 cohort have successfully graduated, and another has submitted their thesis, which is currently under examination. Eight students were enrolled in the second cohort (2024), and five scholarships have been offered for the 2025 cohort, three of which were accepted. Graduates from the first cohort have actively contributed to RECs and various research ethics spaces. They have published two articles, received promotions and new work opportunities linked to their SARETI studies in the region and in the United Kingdom, and presented their work at local and international research ethics and bioethics conferences. Two graduates of SARETI IV have been included as faculty in the current program. The program successfully conducted its inaugural capstone assessment, involving a mock REC activity, which allowed students to apply their theoretical knowledge in a practical setting. Additionally, the program has achieved gender representativity, which enriches the relevance and impact of the training environment. With the focus on early career students, SARETI IV has attracted younger scholars, potentially contributing to better age and gender capacity on RECs. Three graduate students have been appointed as honorary research fellows in the School of Law, UKZN.

##### Challenges

Expanding SARETI IV to include lawyers on RECs was a pivotal innovation for the program. However, the program's historical focus on public health and social sciences has made it challenging to attract legal professionals through previously employed recruitment strategies. Incorporating varied legal frameworks reflective of the continent has been difficult, due to the limited number of trained legal scholars interested in research ethics. Additionally, the heterogeneity of students’ prior educational backgrounds and fields of study has resulted in varying levels of critical thinking, analytical abilities, and writing skills.

##### Solutions

To successfully recruit suitable candidates from the legal field, the program must focus on raising awareness among law faculties across Africa, and RECs with legal members. This will require additional engagement, outreach, and advocacy efforts, clearly communicating the program's relevance and value to legal scholars. To broaden teaching on legal frameworks, SARETI IV is exploring the value of using our own legal graduates to enhance legal training in the program. To address skill gaps among students, the program offers tailored workshops and individualized support, helping students develop critical thinking, improve academic writing, and strengthen time management. Monthly virtual meetings between the SARETI IV Executive and students provide continuous support, enabling real-time problem-solving and ensuring students stay on track. These strategies have mitigated delays, supported student success, and ensured the program meets its objectives effectively.

### ARESA

#### Successes

The ARESA program has successfully graduated 40 students from 10 African countries, many of whom have established careers in health-related professions. The graduates have first- or co-authored 16 research ethics publications in peer-reviewed journals based on their ARESA program research and scholarship. The program has established the annual ARESA seminar, which is attended by professionals in research ethics and bioethics. Additionally, some ARESA graduates have enrolled in the ARESA Leadership PhD program, with two graduates to date (see Ulrich et al., 2025, this issue). The ARESA newsletter has been published 12 times, maintaining engagement among ARESA faculty and graduates.

#### Challenges

As with other African programs, a challenge was the logistics of bringing students from various parts of southern Africa to Stellenbosch, suggesting that incorporating more hybrid or remote learning options could have been beneficial. Another challenge was the critical thinking skills and conceptual analytic abilities of students entering the program, which were sometimes lacking and difficult to improve in a short time. This was reflected in the quality of writing in bioethics and research ethics, which often required significant effort from supervisors or mentors.

#### Solutions

To address these challenges, future programs might consider extending the duration of the program to two years to allow more time for developing critical skills. Additionally, funding for language editing services or software could help improve the quality of academic writing, especially for students for whom English is not their first language. It would also be beneficial to link funding to the creation of academic posts in research ethics or bioethics within their home institutions, ensuring sustainability and capacity building. Guidance on the ethical use of generative artificial intelligence tools and research integrity should be provided from the outset to support student's academic and professional development.

### Zimbabwe

The US-Zim RETP program is one of the newest in the region having been awarded in April 2024. Despite this, the program has already made strides in developing research ethics capacity in Zimbabwe since the country faces challenges such as limited availability of trained faculty and the need for more robust infrastructure. In 2024, five faculty from the University of Zimbabwe attended the Bioethics Intensive program held at George Washington University. This program aimed at strengthening their abilities to develop and deliver bioethics courses at the University of Zimbabwe. Solutions include ongoing faculty development, enhancing online learning platforms, and fostering collaboration with regional and international institutions.

### Botswana, Uganda and Zambia

#### Successes

The FABTP and African Bioethics Consortium has had a deep impact on all member-institutions, providing a mechanism to advance “South-North-South” networking, leadership development, collaborative research, and the sharing of strategies and resources for strengthening ethics capacity at an institutional level. For example, when the consortium was launched, all member-institutions expressed a strong desire to work together to strengthen the quality and efficiency of their IRBs/RECs. Various ideas were discussed at annual consortium meetings and formed the basis of a successful application for a FIC administrative supplement to develop a novel IRB/REC needs assessment tool and pilot it in Zambia and Botswana (Grant #3R25TW001604-13S1). Each institution was also dedicated to the creation of a bioethics center or unit and was committed to providing bioethics-related programming in the form of regular workshops and training for students, faculty and researchers. Through the consortium, detailed “bioethics strategic plans” were also created and implemented at each institution. The consortium additionally helped support partners as they independently submitted grant applications to start three additional programs in research ethics funded by FIC and the European & Developing Countries Clinical Trials Partnership (EDCTP), respectively.

#### Challenges

African Bioethics Consortium collaborators and trainees faced many competing commitments at their home institutions. The efforts of the consortium were but one engagement among a long list of institutional responsibilities that PIs had at their respective universities. Some trainees faced challenges completing their practicum projects in a timely manner. This seemed to be due to a combination of competing priorities and, for some, having to engage in a task—like writing bioethics manuscripts—that they had not done previously in their professional lives.

#### Solutions

The program identified a series of strategies, including clearly designating local mentors and more regular and directed meetings with JHU faculty and staff to provide continued support. Month-long training opportunities were also offered to senior leadership from partner institutions to ensure that those with oversight and management responsibilities had increased awareness of bioethics and were better integrated in coordination of bioethics activities within the institution.

### DRC and Madagascar

#### Successes

Nineteen students completed the MPH with a bioethics concentration at the KSPH in two cohorts (2011 and 2014). A dedicated bioethics unit was established at the KSPH by the scholars supported by the program. Numerous bioethics and research ethics workshops and seminars were conducted in the DRC, including Kinshasa, Goma, and Lubumbashi, as well as in Antananarivo and Mahajanga (Madagascar) and Bujumbura (Burundi). DRC scholars supported by our program contributed to the establishment of the first national research ethics guidelines, and successfully applied for two EDCTP research ethics grants. With project mentorship, three DRC scholars obtained PhD degrees in bioethics-related fields: two from the University of Kinshasa and one from the University of Liege (Belgium). French-language bioethics curriculum materials were developed on a website hosted by the University of North Carolina.

#### Challenges

Together with leadership at the KSPH, a decision was made in 2015 to terminate the MPH (Bioethics) program and integrate bioethics elements into the existing MPH concentrations, after recognizing that KSPH would have insufficient resources to support the program beyond the NIH funding period. Activities in Burundi and plans to expand to the Central African Republic were curtailed by civil unrest. During workshops and seminars, it became clear that many trainees had knowledge gaps about global standards of scientific research, which necessitated allocating extra time and resources to science capacity building. Bioethics-related positions are rare in DRC/Madagascar and trainees faced challenges reconciling bioethics interests with pressing economic needs. The dominance of English-language bioethics literature posed training and curriculum development challenges for French-language speakers.

#### Solutions

If pursuing institutional bioethics capacity-building, program goals need to be closely tied to local and global institutional priorities and resources as these evolve over time. In some contexts, Masters-level degree programs may need to be preceded by the piloting of individual courses and close mentorship of participating faculty. Workshops and seminars can be more impactful if they are integrated into ongoing institutional collaborations. French-language materials on biomedical and social science research design and conduct, highlighting areas that commonly raise ethical issues, would be helpful. Increased efforts to make local authorities aware of how bioethics can contribute to institutional aims could help increase trainee employability. French-language bioethics literature has increased since the project's start, but could gain visibility if centralized and organized in an online platform.

#### Best Practices

These capacity-building programs in health research ethics demonstrate several best practices in research ethics training, namely:

1. Incorporating contextualized training into education can enhance programs by including cultural sensitivity (see ARESA, SARETI and US-Zim RETP) and local ethical-legal standards (Krubiner et al., 2022). For example, in SARETI IV, scholars assess their countries’ policies against international norms, promoting an understanding of the balance between global and local practices. This approach also aids in evaluating health research protocols across different sociocultural backgrounds (Schoeman, 2019).
2. Experiential learning is widely acknowledged as a pivotal element in the consolidation of theoretical knowledge (Gentry, 1990). Several programs adopted this approach by combining theoretical knowledge with hands-on practical experience through assignments, observation of REC meetings, and field excursions to research facilities. By intertwining theoretical frameworks with practical experiences, these programs enhance scholars’ abilities to meaningfully engage with ethical issues in the real world.
3. Several programs incorporated a multidisciplinary approach, integrating social sciences, law, public health, moral and political philosophy, and bioethics. This practice expands the scope of training so that scholars are equipped to review diverse research protocols, addresses an important gap in terms of complex protocols tabled at RECs (Chaundry et al., 2022), and supports the intrinsic multidisciplinarity that characterizes international research ethics activities and leadership.
4. A key best practice from the programs is the emphasis on building networks through seminars, workshops, and peer support groups. These networks foster ongoing engagement and sustainable capacity building by connecting research ethics professionals across the region, and beyond.
5. Student evaluations suggest that personalized coaching supported them in identifying and addressing the non-academic barriers hindering their research and/or career development.

## Research Agenda

These programs contribute to a developing and ongoing health research agenda as scholars focus on conducting empirical or conceptual research of local and global relevance. Key remaining areas for further research include:

1. Ethics of emerging technologies: Given the accelerated global progress in AI, genomics, and big data analytics, the research agenda must urgently include the ethical considerations of these technologies in the international and African context. Understanding REC perspectives on these developments and their capacity to review protocols of emerging technologies is critical.
2. Ethics in public health emergencies: Public health emergencies like Ebola and COVID-19, raised critical ethical issues internationally, regarding informed consent, resource allocation, and the rapid review of research protocols. Future research is needed on flexible and adaptable ethics frameworks that can be used to respond to such emergencies while protecting the welfare of research participants.
3. Trainee tracking: A a comprehensive trainee database is kept by FIC; this should be accessed to track the medium and longer-term accomplishments and placements of trainees to empirically determine program impact and outcomes, including trainees’ research ethics publications and other related outputs. ​

## Educational Implications

We believe that these programs have all innovatively addressed gaps in research ethics capacity. The following educational implications for future programs are noted:

1. Internship opportunities offer practical training with local RECs, exposing trainees to real-time ethics review of research projects, including post-approval compliance monitoring. Internships could also be arranged with leading health research institutes (e.g., CAPRISA, AHRI, SAMRC or the HSRC in South Africa) for exposure to ethical global health research.
2. Programs like the US-Zim RETP and ARESA emphasize the importance of short-term workshops, seminars, and conferences. Postdoctoral fellows and PhD students can participate in or lead such training programs, focusing on current bioethics challenges, public health research, and ethics in clinical trials. These workshops could also be integrated with mentorship programs.
3. Mentorship plays a critical role in advancing careers in health research ethics. Programs like SARETI and ARESA have built-in mentorship and networking opportunities that connect students with global leaders in bioethics. Mentorship initiatives could be extended to PhD and postdoctoral candidates, offering coaching, peer support, and opportunities to engage with leading ethics review committees internationally (cf. e.g., Munro et al., 2024).

## Conclusion

The FIC bioethics training programs have significantly contributed to the strengthening of ethical research capacities in southern Africa and Africa more broadly, extending the accomplishments described in Ndebele et al. (2014a). This significantly improves Africa's ability to contribute to the conduct of ethical, locally and globally relevant health research. By focusing on the specific needs and contexts of South Africa, Zimbabwe, Zambia, the DRC and Madagascar, these programs ensure that health research in the region is conducted with the highest global ethical standards without violating local cultural norms. The ongoing development and enhancement of these programs are crucial as research complexities evolve, incorporating new technologies and responding to emerging local and global health challenges (cf. Abdool-Karim et al., 2017).
